# Effectiveness of self-help workbook intervention on quality of life in cancer patients receiving chemotherapy: results of a randomized controlled trial

**DOI:** 10.1186/s12885-021-08333-2

**Published:** 2021-05-22

**Authors:** Toshimi Takano, Ayako Matsuda, Noriko Ishizuka, Yukinori Ozaki, Koichi Suyama, Yuko Tanabe, Yuji Miura, Eisuke Matsushima

**Affiliations:** 1 Department of Medical Oncology, Toranomon Hospital, 2-2-2 Toranomon, Minato-ku, Tokyo, 105-8470 Japan; 2 Section of Liaison Psychiatry and Palliative Medicine, Graduate School of Medical and Dental Sciences, Tokyo Medical and Dental University, Tokyo, Japan; 3 Department of Hygiene and Public Health, School of Medicine, Teikyo University, Tokyo, Japan; 4 Kumamoto University Hospital Cancer Center, Kumamoto, Japan

**Keywords:** Breast cancer, Chemotherapy, Colorectal cancer, Gastric cancer, Lung cancer, Patient-reported outcome, Psycho-oncology, Psychosocial support system

## Abstract

**Background:**

A self-help workbook is expected to support cancer patients to cope with physical and psychosocial distress, to facilitate communication with medical staff, and to improve quality of life (QOL). We conducted a randomized controlled trial to evaluate the effectiveness of a self-help workbook intervention on QOL and survival.

**Methods:**

From June 2014 to March 2015, patients with breast, colorectal, gastric, and lung cancer receiving outpatient chemotherapy were randomized into an intervention group (*n* = 100) or control group (*n =* 100). Intervention group participants received workbooks originally made for this study, read advice on how to cope with distress, and filled out questionnaires on the workbooks periodically. EORTC QLQ-C30 was evaluated at baseline, at 12 weeks, and at 24 weeks. The primary endpoint was Global Health Status / QOL scale (GQOL).

**Results:**

No significant interaction was observed between the intervention and time in terms of GQOL or any of the functional scales. Among the 69 patients who continued cytotoxic chemotherapy at 24 weeks, the intervention was significantly associated with improved emotional functioning scores (*P* = 0.0007). Overall survival was not significantly different between the two groups.

**Conclusions:**

Self-help workbook intervention was feasible in cancer patients receiving chemotherapy. Although the effect of the intervention was limited, a post-hoc subset analysis suggested that the intervention may improve emotional functioning among patients who receive long-term cytotoxic chemotherapy.

**Trial registration:**

UMIN Clinical Trials Registry, UMIN000012842. Registered 14 January 2014.

## Background

Cancer patients receiving chemotherapy experience the physical and psychosocial distress of coping with a life-threatening disease and treatments that negatively influence their quality of life (QOL) [1]. The number of patients who receive chemotherapy on an outpatient basis is increasing, but few receive enough psychosocial support to relieve their distress.

For cancer patients, mainly for newly diagnosed patients, various psychosocial support programs have been developed and used all over the world, which are based on cognitive behavioral therapy, coping skills training, psychoeducation, psychotherapy, counseling, peer support, and relaxation. Via randomized controlled trials, several researchers have evaluated the effectiveness of such psychosocial support programs, including patient navigation [2], nurse navigator intervention [3], psychoeducational intervention [4], computer-based patient support systems [5], telephone therapy [6], internet peer support [7], internet coping group program [8], expressive writing intervention [9], and meaning-centered group psychotherapy [10], but only a few studies have demonstrated significant effectiveness of these programs. Several meta-analyses have been performed to evaluate the interventions; in two meta-analyses for patients with newly diagnosed early stage cancer, no significant effects were observed for general QOL, while a small improvement was observed when QOL was evaluated using cancer-specific subscales [11] or an emotional subscale [12]. Additionally, in a large-scale meta-analysis for patients with both early and advanced cancer, significant small-to-medium effects on emotional distress and QOL were observed [13]. These data mean that psychosocial support programs are effective in some patients, especially with regard to emotional distress, but the effects are limited and show inconsistent results among all studies and all patients. Considering the cost, limited human resources and limited effects, we need to establish more effective, more efficient, and individualized interventions with better cost-benefit ratios.

Whether or not psychosocial support programs improve overall survival has been another important topic since one study showed survival benefit from supportive group therapy in patients with metastatic breast cancer in 1989 [14]. A meta-analysis showed that psychosocial interventions improved survival at 12 months but not at longer-term follow-up in patients with metastatic breast cancer [15]. In 2010, early palliative care, including psychosocial support, reportedly yielded survival benefit in patients with metastatic non-small-cell lung cancer [16], and now many oncologists recognize the value of early intervention for cancer patients.

A self-help workbook is a simple intervention tool that requires little human and financial resources. It is expected to support patients to cope with distress, to facilitate communication with medical staff, to help decision making, and to improve QOL. Some studies have suggested the effectiveness of a self-help workbook in patients with early breast cancer [17, 18].

We made an original self-help workbook for this study and conducted a randomized controlled trial to evaluate the feasibility of self-help workbook intervention and its effectiveness on QOL. We also exploratorily evaluated overall survival in this study.

## Methods

### Study design

This is an open-label, single-center, randomized trial designed to evaluate QOL in cancer patients treated with standard chemotherapy and supportive care with and without self-help workbooks.

### Patient population

Adult (≥20 years old) patients with breast, colorectal, gastric, or lung cancer who were continuing or started to receive chemotherapy on an outpatient basis at the Department of Medical Oncology, Toranomon Hospital, and who provided written informed consent, were considered eligible for study participation. Patients were excluded if planned duration of their chemotherapy was less than 12 weeks, if they received investigational treatment, if their general condition was regarded as not good enough to allow for participation in this study, if they were unable to read or complete questionnaires in Japanese, or if they had severe cognitive dysfunction or severe psychiatric disorder.

### Randomization

Enrolled patients were randomized to the control arm or the intervention arm on a 1:1 basis using a permuted-block technique using a randomization list with a block size of four.

### Intervention

In the control arm, patients were treated with standard chemotherapy and supportive care without workbooks. In the intervention arm, patients received self-help workbooks written in Japanese. Investigators including medical oncologists, psychiatrists, and clinical psychologists made the workbook originally for this study through discussion while referring to a Japanese book for general readership [19]. The intervention had not been tested in cancer patients before this study.

The workbook comprised two parts. Part one contained seven points of advice on:

1. how to learn about and understand their own disease and condition,
2. how to understand standard care,
3. how to cope with problems related to disease or treatment,
4. how to collect medical information,
5. how to communicate with medical staff,
6. how to make decisions, and
7. how to create their own goals.

Part two contained questionnaires asking patients what their own goals and therapeutic goals were, what their priorities were, and what they wanted to ask medical staff at the next available opportunity. The patients in the intervention arm received the self-help workbooks soon after randomization, and then they read the advice and filled out the questionnaires periodically. At 12 weeks and at 24 weeks, how useful the patients thought the workbooks were and how they used the workbooks were assessed by a checklist.

### Endpoints

The European Organization for Research and Treatment of Cancer (EORTC) Quality of Life Questionnaire-Core 30 (QLQ-C30) was evaluated at baseline, at 12 weeks, and at 24 weeks. The primary endpoint was Global Health Status / QOL scale (GQOL), and the secondary endpoints included five functional scales from the EORTC QLQ C30, i.e., physical, emotional, social, cognitive, and role functioning. Also evaluated and used as adjustment factors were nine symptoms scales, i.e., fatigue, nausea and vomiting, pain, dyspnea, insomnia, appetite loss, constipation, diarrhea, and financial difficulties. All scores were calculated and transformed to a 0–100 scale according to EORTC methods [20]. For GQOL and the functional scales, higher scores represent a higher level of functioning, and for the symptom scales, higher scores represent a greater number of symptoms or difficulties. Overall survival among patients with metastatic disease was also evaluated as an exploratory endpoint.

### Sample size

We did not perform a formal power analysis to calculate the sample size in this study because we could not predict the effect size of self-help workbook intervention and this study was not intended to be confirmatory. Instead, we decided the sample size as 200 randomized patients (100 in each arm) referring to previous randomized controlled trials, in which sample sizes were calculated to detect meaningful differences with a two-sided significance level of 0.05 and power of 0.80.

### Statistical analyses

This randomized controlled trial was designed to evaluate the effects of workbook intervention on GQOL and the functional scales. The primary endpoint was GQOL analyzed in the intention-to-treat (ITT) population. Change of GQOL and functional scales over time (at baseline, at 12 weeks, and at 24 weeks) between the control arm and the intervention arm were evaluated using a mixed effects model. The following covariates were entered into the model as adjustment factors: age, sex, primary site, metastatic disease, employment status, marital status, and changes in symptom scales from baseline to 24 weeks. In the analyses of the ITT population, missing data were handled using ignorable maximum likelihood estimation. We also performed a post-hoc subgroup analyses among patients who continued cytotoxic chemotherapy at 24 weeks because the workbook was directed at patients receiving cytotoxic chemotherapy. Because scores of GQOL and the functional scales can increase and decrease during cancer treatment, we focused on the differences of QOL changes between the two arms, and the interactions between the intervention and time were mainly evaluated rather than the improvement or deterioration of QOL in each arm.

As an exploratory analysis, overall survival was compared between two arms using Kaplan–Meier analysis with log-rank tests. Statistical analyses were performed using SPSS Statistics 24 (IBM Corporation, Armonk, NY).

## Results

**Fig. 1:**
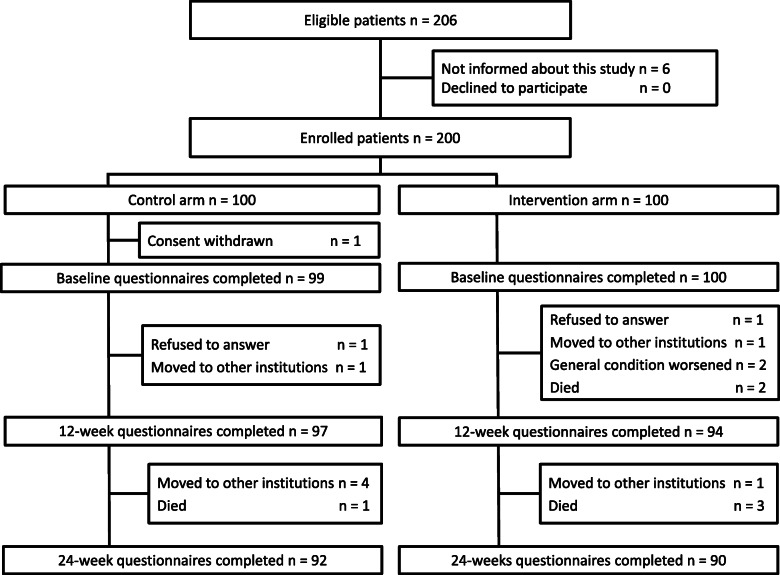
Consolidated Standards of Reporting Trials flow diagram of the current study. GQOL, global health status / quality of life scale

From June 2014 to March 2015, 200 patients among 206 eligible patients were enrolled and randomized to the control arm (*n* = 100) or the intervention arm (*n =* 100). One patient who was randomized to the control arm withdrew consent before answering the first questionnaire at baseline and was excluded from all analyses. The other 199 patients were included in the ITT analysis. Ninety-seven patients (98%) in the control arm and 94 patients (94%) in the intervention arm completed the questionnaires at 12 weeks, and 92 patients (93%) in the control arm and 90 patients (90%) in the intervention arm completed them at 24 weeks (Fig. 1). Among the completed questionnaires, some items of the EORTC QLQ C30 were unanswered by 4 patients (4%) in the control arm and 6 patients (6%) in the intervention arm. In the intervention arm, 96 patients (96%) completed checklists at 12 weeks and 83% of them answered that the workbooks were useful.

Among the ITT population, 69 patients (35%) continued cytotoxic chemotherapy at 24 weeks. Others discontinued cytotoxic chemotherapy due to completion of planned treatment, deterioration of their general condition, or upon their own request. Baseline characteristics of the ITT population and the patients who continued cytotoxic chemotherapy at 24 weeks are shown in Table 1. Among the patients who continued cytotoxic chemotherapy at 24 weeks, younger patients, those without metastatic disease, and those not previously treated with chemotherapy were more in the intervention arm.

**Table 1:** Baseline characteristics of the intent-to-treat polulation and the patients who continued chemotherapy at 24 weeks

	Intent-to-treat population (*n* = 199)			Patients who continued cytotoxic chemotherapy at 24 weeks (*n* = 69)		
	Control	Intervention	*P*^a^	Control	Intervention	*P*^a^
Total, n	99	100		38	31	
Age, years			0.427			0.044
Median	60	57		62	53	
Range	33–83	28–77		33–82	28–73	
Sex, n (%)			0.450			0.328
Male	21 (21%)	17 (17%)		14 (37%)	8 (26%)	
Female	78 (79%)	83 (83%)		24 (63%)	23 (74%)	
Primary site, n (%)			0.909			0.444
Breast	66 (67%)	70 (70%)		16 (42%)	17 (55%)	
Colorectal	24 (24%)	21 (21%)		15 (39%)	12 (39%)	
Gastric	6 (6%)	5 (5%)		5 (13%)	1 (3%)	
Lung	3 (3%)	4 (4%)		2 (5%)	1 (3%)	
Metastatic disease, n (%)			0.807			0.059
Yes	65 (66%)	64 (64%)		32 (84%)	20 (65%)	
No	34 (34%)	36 (36%)		6 (16%)	11 (35%)	
Prior chemotherapy, n (%)			0.251			0.061
Yes	80 (81%)	74 (74%)		31 (82%)	19 (61%)	
No	19 (19%)	26 (26%)		7 (18%)	12 (39%)	
Employment status, n (%)			0.817			0.458
Employed	39 (39%)	41 (41%)		15 (39%)	15 (48%)	
Not employed	60 (61%)	59 (59%)		23 (61%)	16 (52%)	
Marital status, n (%)^b^			0.799			0.669
Married	81 (82%)	84 (84%)		31 (82%)	24 (77%)	
Not married	17 (17%)	16 (16%)		7 (18%)	7 (23%)	
Education level, n (%)^c^			0.545			0.952
High school	34 (34%)	31 (31%)		12 (32%)	10 (32%)	
College or more	63 (64%)	69 (69%)		26 (68%)	21 (68%)	

^a^T-test was used to examine differences in attribution for age between the two arms. Chi-square tests were performed to examine differences in attribution for other factors

^b^Marital status was unknown in one patient in the control arm of Intent-to-treat population

^c^Education level was unknown in two patients in the control arm of Intent-to-treat population

**Fig. 2:**
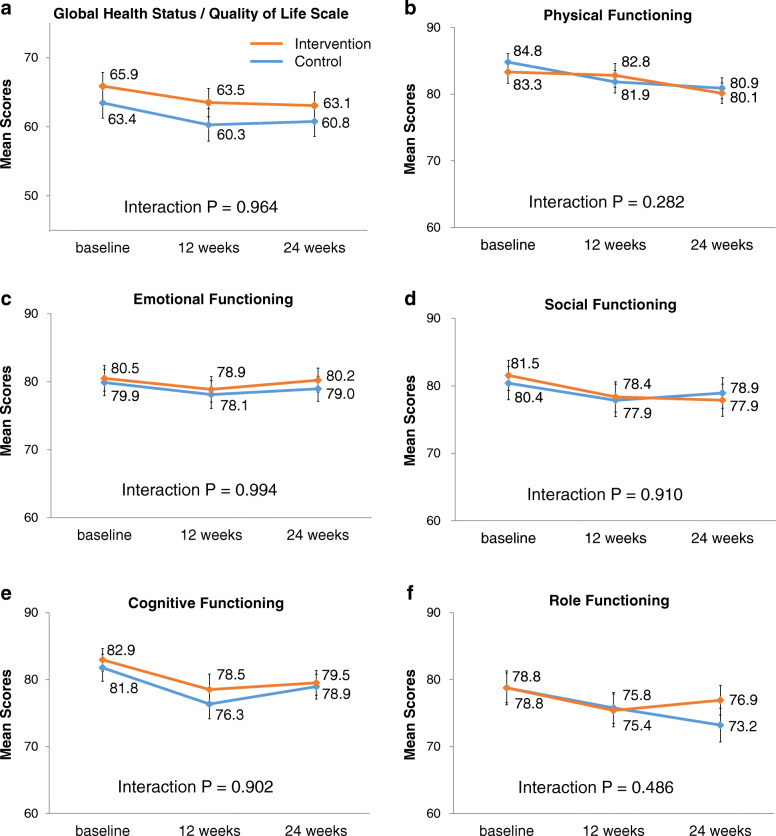
Changes in the EORTC QLQ-C30 scores in the intent-to-treat population. Changes in the European Organization for Research and Treatment of Cancer (EORTC) QLQ-C30 scores in the intent-to-treat population (*n =* 199): **a** Global Health Status / Quality of Life scale (GQOL), (**b**) physical functioning, (**c**) emotional functioning, (**d**) social functioning, (**e**) cognitive functioning, and (**f**) role functioning. All scores were calculated and transformed to a 0–100 scale according to EORTC methods. Higher scores represent a higher level of functioning. Missing data were handled using ignorable maximum likelihood estimation

Among the ITT population, changes in mean scores of GQOL and the functional scales in the two arms are shown in Fig. 2. At baseline, at 12 weeks, and at 24 weeks, mean scores (standard deviation) of GQOL were 63.4 (22.0), 60.3 (23.4), and 60.8 (21.5) in the control arm and 65.9 (19.8), 63.5 (20.5), and 63.1 (19.6) in the intervention arm. No significant interaction was observed between the intervention and time in terms of GQOL (*P* = 0.964) or any of the functional scales.

**Fig. 3:**
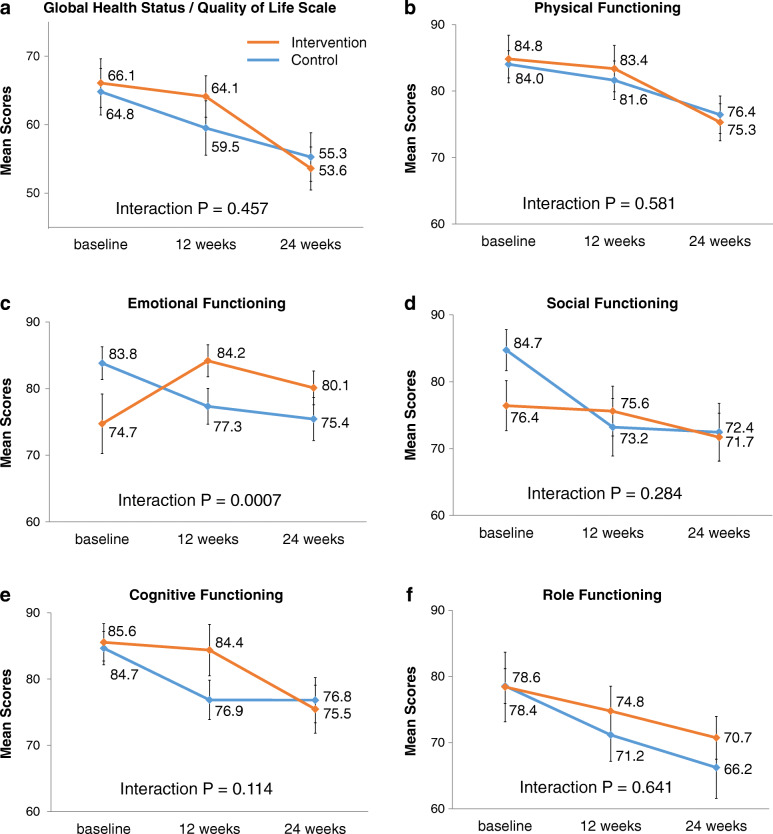
Changes in the EORTC QLQ-C30 scores in patients who continued cytotoxic chemotherapy at 24 weeks. Changes in the European Organization for Research and Treatment of Cancer (EORTC) QLQ-C30 scores in patients who continued cytotoxic chemotherapy at 24 weeks (*n =* 69): **a** Global Health Status / Quality of Life scale (GQOL), (**b**) physical functioning, (**c**) emotional functioning, (**d**) social functioning, (**e**) cognitive functioning, and (**f**) role functioning

The changes of mean scores of GQOL and the functional scales in the 69 patients who continued cytotoxic chemotherapy at 24 weeks are shown in Fig. 3. Although most scores tended to worsen over time, only emotional functioning scores in the intervention arm showed improvement at 12 weeks and at 24 weeks than at baseline. Significant interaction was shown between the intervention and time on emotional functioning (*P* = 0.0007). The intervention was not significantly associated with changes of GQOL and other functional scales than emotional functioning.

The median follow-up time was 37.3 months among patients with metastatic disease. Up to 33 of 65 patients in the control arm and 28 of 64 patients in the intervention arm died, and overall survival was not observed to be significantly different between the two arms (median, 30.4 months in the control arm and not reached in the intervention arm; hazard ratio, 0.832; 95% confidence interval, 0.499–1.376; log-rank *P* = 0.474).

## Discussion

This randomized controlled trial showed no significant improvement in GQOL and overall survival; however, a post-hoc subset analysis suggested that the intervention improved emotional functioning among patients who received long-term cytotoxic chemotherapy.

In this study, we used self-help workbooks, which are a simple intervention tool that requires little human and financial resources, and showed that intervention was feasible in cancer patients receiving chemotherapy on an outpatient basis. The intervention was expected to support patients to cope with distress, to facilitate communication with medical staff, to help decision making, and to improve QOL.

Some previous studies have suggested that psychosocial support programs are effective in treating emotional distress, although they showed limited effects on general QOL [12, 13], and the current results were consistent with these prior findings. Although the workbook intervention was not specific to emotional functioning, we consider that facilitation of communication and shared decision making with medical staff was possibly effective to improve emotional functioning among patients receiving long-term cytotoxic chemotherapy, who often feel anxious about the treatment and their prognosis.

The workbook intervention may have been too simple to show significant improvement in GQOL scales. Most psychosocial support programs require participation of experts who intervene in patients periodically thorough one-on-one or face-to-face contact. Though such programs are beneficial in some patients, they are costly and do not consistently appeal to all patients. In such programs, individualization is important; however, this depends on the ability of the experts and flexibility of the programs. In our workbook intervention, patients received workbooks, and by reading and writing in the workbooks, they were prompted to set their own goals and informed of the resources they could access to achieve their goals. When every patient can make decisions and take actions proactively, this approach will be more effective; however, the workbook intervention might be too weak to make substantial differences.

This study has several limitations. First, as mentioned above, the workbook intervention might be weak because what we did was merely give workbooks to patients without direct interventions by experts. We may be able to increase the effectiveness of the intervention by using workbooks more systematically or by using newer tools.

Second, the subgroup analyses among patients who continued cytotoxic chemotherapy at 24 weeks were post-hoc, the number of patients included in the subgroup was relatively small, and there were imbalances in baseline characteristics such as age, metastatic disease, and prior chemotherapy between the arms. Although we included age and metastatic disease as adjustment factors in the analysis, it cannot be denied that the result of the improvement of emotional functioning was caused by the imbalances or by chance. Thus, the result should be interpreted as exploratory.

Third, interpretation and extrapolation to other situations are difficult because this study was conducted at a single institution and included a diverse population with breast, colorectal, gastric and lung cancer, with and without metastatic disease. We enrolled various cancer patients because this study was the first one to evaluate the feasibility of the current workbook. Since the majority were patients with breast cancer (68%) and females (81%), results can be influenced by this distribution; however, we could not identify the difference between breast cancer and other cancers and between females and males in this study. As a next step, we need multi-institutional trials with more effective interventions in more specific patients.

Based on the results with a simple prototype program, we are planning to develop more refined programs and to conduct studies to evaluate them. To enhance the effectiveness of interventions, we think that we should make the best use of web-based systems. Recently, some web-based self-management interventions have been studied in cancer patients or cancer survivors. The Breast Cancer E-Health (BREATH) trial showed that a web-based self-management intervention significantly reduced distress in early breast cancer survivors [21], and another study suggested that a web-based self-management intervention called RESTORE may enhance self-efficacy to manage cancer-related fatigue in cancer patients after curative-intent treatment [22].

Web-based systems can also facilitate communication between cancer patients and medical staff. A recent randomized controlled trial showed that systematic web-based collection of patient-reported symptoms improved health-related QOL and overall survival in patients with advanced solid tumors receiving outpatient chemotherapy [23, 24]. These results and recent development of technology encourage us to make more effective and efficient tools for cancer patients.

## Conclusions

Self-help workbook intervention was feasible in cancer patients receiving outpatient chemotherapy. Although the effect of the intervention on QOL was limited, a post-hoc subset analysis suggested that the intervention may improve emotional functioning among patients who receive long-term cytotoxic chemotherapy.

## Data Availability

The datasets used and/or analyzed during the current study are available from the corresponding author on reasonable request.

## Abbreviations

QOL: Quality of life
EORTC: European Organization for Research and Treatment of Cancer
QLQ-C30: Quality of Life Questionnaire-Core 30
GQOL: Global Health Status / QOL scale
ITT: Intention-to-treat
